# Biochemical and Anthropometric Outcomes in Paediatric Patients with Heterozygous Familial Hypercholesterolemia after COVID-19 Pandemic Lockdowns: An Exploratory Analysis

**DOI:** 10.3390/nu16132170

**Published:** 2024-07-08

**Authors:** Maria Peres, André Moreira-Rosário, Gonçalo Padeira, Patrícia Gaspar Silva, Carla Correia, Andreia Nunes, Elisabete Garcia, Ana Faria, Diana Teixeira, Conceição Calhau, Luís Pereira-da-Silva, Ana Cristina Ferreira, Júlio César Rocha

**Affiliations:** 1 Nutrition and Metabolism, NOVA Medical School, Faculdade de Ciências Médicas, Universidade NOVA de Lisboa, 1169-056 Lisboa, Portugal; mariacpperes@gmail.com (M.P.); andre.rosario@nms.unl.pt (A.M.-R.); ana.faria@nms.unl.pt (A.F.); diana.teixeira@nms.unl.pt (D.T.); ccalhau@nms.unl.pt (C.C.); 2 CHRC—Comprehensive Health Research Centre, Nutrition Group, NOVA Medical School, Faculdade de Ciências Médicas, Universidade NOVA de Lisboa, 1169-056 Lisboa, Portugal; l.pereira.silva@nms.unl.pt; 3 CINTESIS—Center for Health Technology and Services Research, NOVA Medical School, 1169-056 Lisboa, Portugal; 4 Reference Centre of Inherited Metabolic Diseases, Unidade Local de Saúde São José, Centro Clínico Académico de Lisboa, 1169-045 Lisboa, Portugal; goncalo.padeira@ulssjose.min-saude.pt (G.P.); patriciacgfsilva@gmail.com (P.G.S.); carla.martins@ulssjose.min-saude.pt (C.C.); andreia.nunes@ulssjose.min-saude.pt (A.N.); elisabete.garcia@ulssjose.min-saude.pt (E.G.); anacristina.ferreira@ulssjose.min-saude.pt (A.C.F.); 5 Medicine of Woman, Childhood and Adolescence Academic Area, NOVA Medical School, Faculdade de Ciências Médicas, Universidade NOVA de Lisboa, 1169-056 Lisboa, Portugal

**Keywords:** anthropometry, COVID-19, familial hypercholesterolemia, LDL cholesterol

## Abstract

The COVID-19 pandemic lockdowns affected the lifestyles of children and adolescents, leading to an increase in childhood obesity. Paediatric patients with familial hypercholesterolemia (FH) may be more susceptible to lockdown effects due to their increased cardiovascular risk. However, data are lacking. We investigated the effect of lockdowns on the metabolic profile of paediatric patients with FH. Blood lipids and anthropometry measured in September 2021–April 2022 were retrospectively compared with pre-pandemic values. Thirty participants were included (1–16 years; 57% female). From baseline to post-pandemic, median [P25, P75] blood LDL-C concentration was 125 [112, 150] mg/dL vs. 125 [100, 147] mg/dL (*p* = 0.894); HDL-C was 58 [52, 65] mg/dL vs. 56 [51, 61] mg/dL (*p* = 0.107); triglycerides were 64 [44, 86] mg/dL vs. 59 [42, 86] mg/dL (*p* = 0.178). The BMI z-score did not change significantly (0.19 [−0.58, 0.89] vs. 0.30 [−0.48, 1.10], *p* = 0.524). The lack of deterioration in metabolic profiles during lockdowns is positive, as some deterioration was expected. We speculate that patients and caregivers were successfully educated about healthy lifestyle and dietary habits. Our results should be interpreted with caution since the study sample was small and heterogeneous. Multicentre research is needed to better understand the impact of lockdowns on this population.

## 1. Introduction

Familial hypercholesterolemia (FH) is an autosomal dominant genetic disorder that affects the metabolism of low-density lipoproteins (LDL), resulting in elevated concentrations of LDL cholesterol (LDL-C) in the blood from birth [1]. Unlike homozygous FH, which is extremely rare, heterozygous FH (or simply FH) affects about one in 300 people [2,3] and is associated with a milder phenotype [4].

It is estimated that 40% of patients with FH have a known genetic cause for this disorder [5], 90% of these due to mutations in the LDL receptor (LDLR) gene [6]. The LDLR is responsible for the uptake of circulating LDL into the liver [7]. Thus, any mutation that impairs the function of this receptor results in deficient LDL-C uptake and its accumulation in the bloodstream [8]. It is estimated that 88% of patients clinically diagnosed with FH without a known genetic cause may have polygenic hypercholesterolemia [9]. This occurs due to the presence of an above average number of alleles that raise LDL-C. [10].

Lifelong cumulative exposure to elevated LDL-C levels severely increases the risk of premature atherosclerosis and cardiovascular disease (CVD) [4,11,12]. Children with FH often show early signs of atherosclerosis [4], and at 25–39 years old, their risk of incident coronary heart disease is 11 and 17 times higher (for men and women, respectively) than the general population [13]. Treatment for FH should therefore be initiated as early as possible to maximize its benefits—preferably during childhood [14]. Lifestyle interventions are the first line of treatment, with the goal of optimizing lipid profiles. Adopting a healthy lifestyle early in life is also key to preventing additional CVD risk factors, such as tobacco use, poor diet, physical inactivity and several chronic illnesses [15,16,17,18]. Many cases require additional treatment with lipid-lowering drugs, such as statins and ezetimibe [4], which can effectively lower blood LDL-C [19] and, ultimately, reduce CVD risk and mortality in adulthood [20,21]. Ideally, treatment should be conducted at a specialized centre [19] by a multidisciplinary team [17], and blood LDL-C should be monitored every 3 to 6 months [22].

Coronavirus disease 2019 (COVID-19) is a highly transmissible respiratory syndrome. It was declared a pandemic in early 2020 due to its rapid global spread [23,24]. As health services came under severe pressure, many countries implemented restrictive measures to contain the spread of COVID-19 [25,26]. In Portugal, the government imposed two national lockdowns (March–May 2020 and January–March 2021), with mandatory home confinement, closure of schools and businesses, and the obligation to work from home. Health services were also affected, with many appointments, surgeries and interventions being delayed or cancelled. Once the lockdowns were lifted, other restrictive measures were gradually withdrawn over the course of several months [27].

These measures were particularly disruptive for children and adolescents, who had to adapt to online classes, isolation from peers and changes in daily routines [28]. Many—especially those with chronic illnesses—experienced worsening of their mental health, with higher levels of stress, anxiety, depression and even suicidal ideation [29,30,31,32,33].

The eating behaviours and physical activity habits of young people were also impacted. Despite conflicting evidence from isolated studies [34,35], one systematic review [36] demonstrated an overall tendency towards healthier eating habits during lockdown. Adolescents and those from lower socioeconomic groups, however, were more likely to adopt worse eating patterns during this time. The Childhood Obesity Surveillance Initiative (COSI) study [37] showed that, despite an increase in the intake of savoury snacks and sweets, eating home-cooked meals as a family became more frequent among primary school age children. Time spent being physically active among children and adolescents also decreased by 20% during the COVID-19 pandemic [38]. Ultimately, the pandemic landscape has led to increases in childhood obesity rates around the world [39,40]. In Portugal, childhood overweight and obesity increased from 2019 to 2022 for the first time in over a decade, which contradicted the decreasing tendency in previous years [41].

To the best of our knowledge, the effects of COVID-19 lockdowns have been studied in adult patients with FH [42,43] but not in paediatric populations. In adults with FH, negative impacts of the COVID-19 lockdowns on dietary habits, physical activity and lipid profile were reported [42,43,44]. Based on these data, we hypothesized that the lipid and anthropometric profiles of paediatric patients with FH deteriorated during the lockdowns caused by the COVID-19 pandemic.

The aim of this study was to determine whether the COVID-19 pandemic lockdowns affected the lipid and anthropometric profiles of paediatric patients with FH in an exploratory analysis.

## 2. Materials and Methods

### 2.1. Study Design

This was a retrospective, longitudinal single-centre study conducted at the Reference Centre of Inherited Metabolic Diseases in Hospital Dona Estefânia, Unidade Local de Saúde São José (ULSSJ).

The participants went through a routine clinical and nutritional assessment between September 2021 and April 2022 (post-pandemic) that included biochemical and anthropometric measurements, which were retrospectively compared with pre-pandemic (baseline) results. We considered the last biochemical analysis performed before the first national lockdown was declared (March 2020).

**Figure 1:**
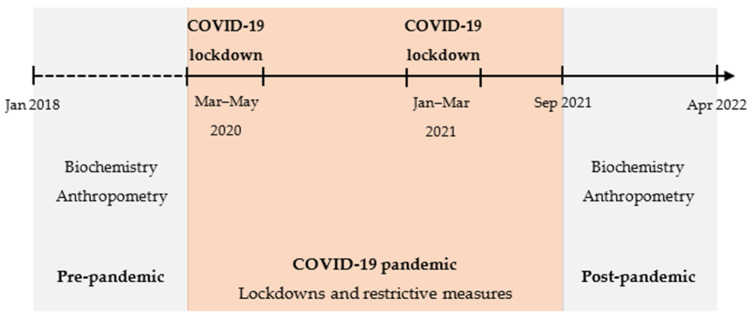
Study design. COVID-19, coronavirus disease 2019.

The primary outcomes were changes in LDL-C concentrations and BMI z-scores between both assessments. Changes in the remaining biochemical and anthropometric parameters were secondary outcomes. The study design is illustrated in Figure 1.

### 2.2. Participants

**Figure 2:**
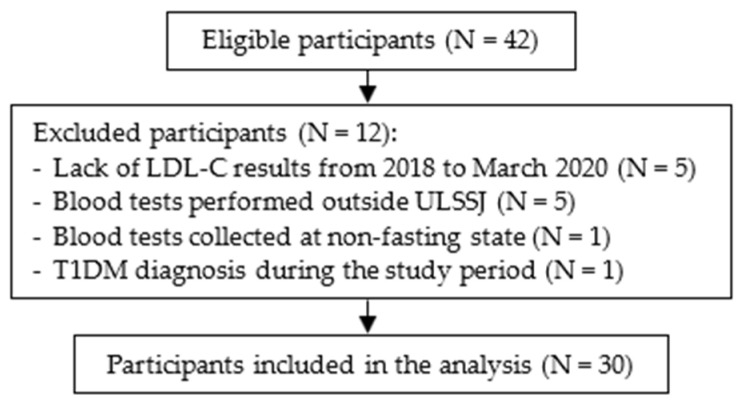
Participant flowchart. ULSSJ, Unidade Local de Saúde São José; LDL-C, low-density lipoprotein cholesterol; T1DM, type 1 diabetes mellitus.

Patients who attended a nutrition appointment between September 2021 and April 2022 were eligible to participate if they were 18 years old or younger, had a confirmed diagnosis of FH and were in follow-up at ULSSJ in 2019. The Simon Broome diagnostic criteria [45] were used to confirm an FH diagnosis. We identified 42 eligible individuals. Exclusion criteria were lack of LDL-C results from 2018 to March 2020 (N = 5); blood tests performed outside of ULSSJ (N = 5); blood samples collected at a non-fasting state (N = 1); and diagnosis of type 1 diabetes mellitus (T1DM) during the study period (N = 1) (Figure 2).

Patients were invited to participate by the clinical team at routine follow-up hospital visits. A convenience sampling method was used. To reduce the risk of bias and obtain a more representative sample, consecutive cases meeting the inclusion criteria were recruited.

### 2.3. Data Collection

Data collection was conducted between February and May of 2022 by retrieving data from hospital clinical records. Gender, date of birth, age at follow-up initiation at the Reference Centre, pharmacological treatment with cholesterol-lowering drugs at baseline, length of follow-up at baseline, pre-treatment LDL-C concentration, genetic testing results, family history in terms of hypercholesterolemia, presence of FH mutations, premature CHD and sudden death were documented. Premature CHD was considered when it occurred before the age of 55 years in men and 60 years in women [46].

#### 2.3.1. Lipid Profile and Other Biochemical Parameters

The following parameters of lipid profile were collected at baseline and post-pandemic: LDL-C, HDL-C, triglycerides (TG), total cholesterol (TC), lipoprotein a (Lp(a)), apolipoprotein A (apoA) and apolipoprotein B (apoB). Additional biochemical parameters included glucose, alanine aminotransferase (ALT) and aspartate aminotransferase (AST). Measurements were performed on blood samples collected after an overnight fast at the laboratory in ULSSJ from the Reference Centre of Local Health Unit São José.

Blood lipid concentrations were classified as acceptable, borderline or abnormal according to the cut-offs established in the 2018 Blood Cholesterol guideline, published by the American College of Cardiology and the American Heart Association [47]. LDL-C blood levels were classified as acceptable (<110 mg/dL), borderline (110–129 mg/dL) or abnormal (≥130 mg/dL). For HDL-C, ranges were >45 mg/dL.

The following blood lipid results were considered acceptable, borderline and abnormal, respectively: for LDL-C, <110 mg/dL, 110–129 mg/dL and ≥130 mg/dL; for HDL-C, >45 mg/dL, 40–45 mg/dL and <40 mg/dL; for TG (0–9 years of age), <75 mg/dL, 75–99 mg/dL and ≥100 mg/dL; for TG (10–19 years), <90 mg/dL, 90–129 mg/dL and ≥130 mg/dL; and for TC, <170 mg/dL, 170–199 mg/dL and ≥200 mg/dL.

#### 2.3.2. Anthropometry

Weight and height were measured in light clothing at baseline and post-pandemic. Body weight was measured used a SECA^®^ electronic scale (GmbH & Co. KG, Birmingham, UK) having a precision of 0.1 kg, and height was measured using a stadiometer to the nearest 1 mm. At baseline, single measurements were performed, while in the post-pandemic timepoint, the average of three measurements was calculated. Body mass index (BMI) was calculated as weight (kg)/height (m)^2^. Weight, height and BMI z-scores were calculated and classified according to the World Health Organization age-specific criteria [48,49,50], using the software WHO Anthro^®^ (WHO, Version 3.2.2) and WHO AnthroPlus^®^ (WHO, Version 1.0.4).

### 2.4. Statistical Analysis

Statistical analysis was performed using the software IBM^®^ SPSS^®^ Statistics 26 for Windows. Statistical significance was considered when *p* < 0.05.

The normality of continuous variable distributions was assessed using the Shapiro–Wilk test, complemented by histogram analysis. Normally distributed continuous variables were presented as mean ± standard deviation (SD), while non-normally distributed variables were reported as the median and interquartile range [P25, P75]. For comparisons within the same group (baseline versus post-pandemic), parametric tests (paired samples Student’s *t*-test) and nonparametric tests (Wilcoxon signed-rank test) were used where appropriate, considering normality assumptions. Hypotheses for categorical variables were tested using a chi-squared test or Fisher’s exact test, as appropriate.

A secondary analysis was conducted to explore potential associations between changes in LDL-C concentrations or BMI z-score and various variables, including biochemical and anthropometric measures, gender, age, genotype and pharmacotherapy. To establish associations with quantitative variables, each variable was categorized into two to three clinically relevant groups, aiming for relatively equal case distribution. Changes in LDL-C concentrations or BMI z-score within each category were assessed using the paired samples Student’s t-test or the Wilcoxon signed-rank test, depending on the normality of the variable’s distribution. Furthermore, differences in changes in LDL-C concentrations and BMI z-score between categories of each variable were analysed. For variables with two categories (e.g., gender), differences were determined using the independent samples t-test for normally distributed variables or the Mann–Whitney test for non-normally distributed variables. For variables with three categories, One-Way ANOVA and Kruskal–Wallis statistical tests were used for normally and non-normally distributed variables, respectively.

### 2.5. Ethical Statement

This research project respected ethical principles and was approved by the Ethical Committees of NOVA Medical School, Universidade NOVA de Lisboa (No. 128/2021/CEFCM) and Unidade Local de Saúde São José (Process No. 1113/2021). Each participant’s parent was asked to give written informed consent.

Data were collected and used in conformity with the General Data Protection Regulation of the European Union (Regulation (EU) 2016/679) to ensure subject confidentiality. Each participant was given a unique identification number to be used in the project database. Participant names and contact information were stored in a separate file. Both documents were protected by a password and were accessible only to clinical and research team members.

## 3. Results

### 3.1. Sample Characteristics

Table 1 shows the characteristics of the study sample (N = 30) at baseline, i.e., in the pre-pandemic period. The study sample included 13 boys and 17 girls aged 1 to 16 years, with a median of 11 years. Participants had been followed up at the Reference Centre for Inherited Metabolic Diseases since the median age of 8 years. In March of 2020, participants had been followed for 3 [1, 5] years (median [P25, P75]).

The median [P25, P75] pre-treatment LDL-C concentration was 158 [136, 197] mg/dL. In most cases, the FH diagnosis was based solely on clinical criteria. Twelve (40%) participants also underwent genetic testing, and three had a confirmed FH causing mutation in the LDLR gene. Three other participants had mutations of likely pathogenicity in the same gene. Five participants had at least one family member with the same mutation. About 27% of participants had a known family history of premature CHD or sudden death, while 90% had family history of hypercholesterolemia.

Changes in LDL-C concentrations or BMI z-score were not associated with any of the previously mentioned variables.

### 3.2. Lipid Profile and Other Biochemical Parameters

For each variable, the results shown in Table 2 refer to participants who had both pre- and post-pandemic data available. Other participants were excluded from this analysis for better comparability.

The median LDL-C concentrations were unchanged between the baseline (125 [112, 150] mg/dL) and post-pandemic period (125 [100, 147] mg/dL); *p* = 0.894. The median concentrations of HDL-C, TG and TC did not change significantly between baseline and the post-pandemic period, as seen in Table 2. However, TC results were available for only a third of participants.

**Figure 3:**
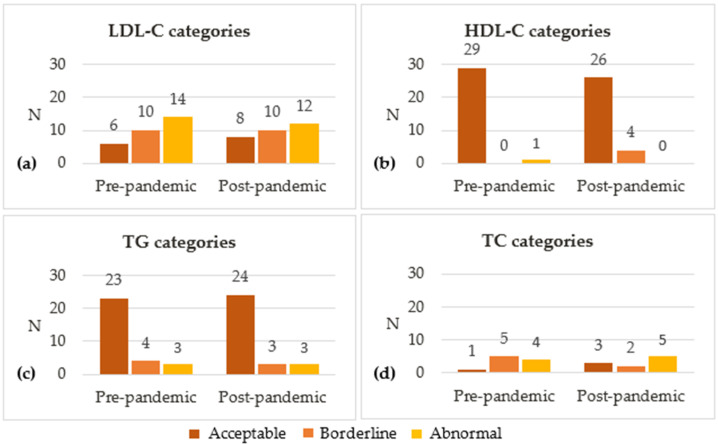
Distribution of different categories of (**a**) LDL-C, (**b**) HDL-C, (**c**) TG and (**d**) TC. The following blood lipid results were considered acceptable, borderline and abnormal, respectively: for LDL-C, <110 mg/dL, 110–129 mg/dL and ≥130 mg/dL; for HDL-C, >45 mg/dL, 40–45 mg/dL and <40 mg/dL; for TG (0–9 years of age), <75 mg/dL, 75–99 mg/dL and ≥100 mg/dL; for TG (10–19 years), <90 mg/dL, 90–129 mg/dL and ≥130 mg/dL; and for TC, <170 mg/dL, 170–199 mg/dL and ≥200 mg/dL. HDL-C, high-density lipoprotein cholesterol; LDL-C, low-density lipoprotein cholesterol; TC, total cholesterol; TG, triglycerides.

Figure 3 shows the classification of blood lipid concentrations as acceptable, borderline and abnormal. Between baseline and post-pandemic, the percentage of participants with acceptable LDL-C blood levels (<110 mg/dL) increased from 20% to 27%, while those with abnormal LDL-C levels (≥130 mg/dL) decreased from 47% to 40%. Most participants had acceptable levels of HDL-C (>45 mg/dL; 97% vs. 87%) and TG (<75 mg/dL or <90 mg/dL, respectively, for those aged <10 and ≥10 years; 77% vs. 80%) on both period assessments. A minority of the subsample whose TC levels were measured had acceptable TC levels (<170 mg/dL; 10% vs. 30%), with 40% to 50% having abnormal concentrations (≥200 mg/dL) at each moment.

Data were missing for other biochemical parameters, mostly at baseline. The available data allowed comparisons between pre- and post-pandemic results in 10 to 19 participants from the total sample, depending on the variable. Concentrations remained almost unchanged for Lp(a), apoA, apoB, glucose, ALT and AST (as seen in Table 2).

No significant associations were found between any other biochemical variables and changes in LDL-C concentrations or BMI z-score between pre- and post-pandemic periods.

### 3.3. Anthropometry

**Figure 4:**
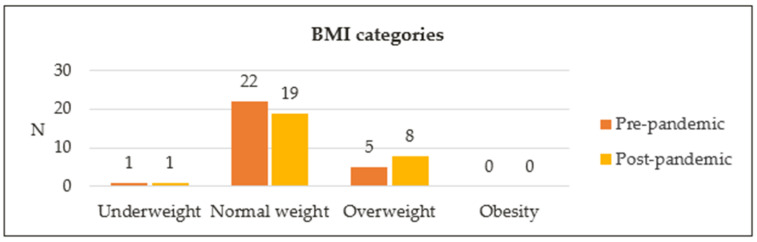
Distribution of different BMI categories. BMI, body mass index.

Twenty-eight participants were included in this analysis, as two children had missing anthropometric data at baseline (Table 2). Significant increases (*p* < 0.001) were seen in median weight, height and BMI between baseline and the post-pandemic period. Changes in height z-score and BMI z-score, however, were not significant. Median height z-score was −0.72 [−1.14, 0.56] at baseline, compared with −0.54 [−1.17, 0.90] post-pandemic (*p* = 0.097). Median BMI z-score increased from 0.19 [−0.58, 0.89] at baseline to 0.30 [−0.48, 1.10] post-pandemic (*p* = 0.524). The distribution of BMI categories is represented in Figure 4. Most participants had normal BMI for their age: 79% at baseline vs. 68% post-pandemic. Five participants (18%) were classified as overweight at baseline, vs. eight participants (29%) post-pandemic. One participant who was overweight at baseline reverted to normal weight post-pandemic, while four others of normal weight became overweight in the post-pandemic period.

No significant associations were found between anthropometric variables and changes in LDL-C concentrations or BMI z-score between the pre- and post-pandemic periods.

## 4. Discussion

The pandemic caused by COVID-19 led to lockdowns, school closures, self-isolation and social distancing measures across the globe [25]. These restrictive measures have impacted the physical and mental health of children and adolescents [31,51,52,53,54], disrupting their dietary habits [35,36], decreasing physical activity and promoting sedentary behaviours [52,55,56]. In particular, a reduction in physical activity may have an independent negative effect on the lipid profile of children and adolescents [57,58,59]. In this exploratory analysis, we investigated the effects of the COVID-19 pandemic lockdowns on biochemical and anthropometric profiles of children and adolescents with FH in follow-up at our Reference Centre of Inherited Metabolic Diseases. We hypothesized that blood lipids and anthropometric profiles would deteriorate between the pre-pandemic period and September 2021 to April 2022. However, the exploratory findings obtained in a sample of 30 paediatric patients with FH did not confirm our hypothesis. No significant changes were observed between the pre- and post-pandemic periods in median blood concentrations of LDL-C, other parameters of lipid profile or remaining biochemical parameters. The median z-scores for height and BMI remained stable, with very small and non-significant increases.

The changes in median LDL-C concentrations were not significant between the two time point assessments, and the slight increase in TC and decreases in HDL-C and TG were neither statistically nor clinically significant. Taken together with steady Lp(a), apoA and apoB, this suggests an overall stable lipid profile of the participants between the pre-pandemic period and September 2021 to April 2022. The distribution of LDL-C concentrations across the different percentile categories (normal, borderline and abnormal) was also similar in the pre- and post-pandemic periods, which further confirms this notion.

Significant lipid profile deterioration has also been documented in adults with [60] and without [61,62,63] dyslipidaemia. Specifically in adults with FH, the SARS-CoV-2 pandemic severely limited access to lipidologists and cardiologists, resulting in a reduction in lipid profile assessments [43,44]. In these patients, lockdown restrictions also promoted sedentary lifestyles, decreased dietary adherence and increased intake of fatty meals [42,44]. In a retrospective study of 260 adults with FH, significant alterations in the lipid profile were observed 12 months after the implementation of the lockdown measures. These findings included a reduction in the mean HDL-C (53.2 vs. 47.8 mg/dL, *p* < 0.05) and an increase in the mean non-HDL-C (117.2 vs. 133.1 mg/dL, *p* < 0.05) [43]. On the other hand, no post-lockdown changes were found in the lipid or glycaemic profiles of children and adolescents with obesity [64,65]—but an increase in the severity of obesity was noted [64].

Regardless of the COVID-19 pandemic lockdowns, some blood lipid profile deterioration could be expected given that the median age of the study population was 11 years at baseline and 13 years in the post-pandemic period. A tendency towards increases in TC, LDL-C and TG during early adolescence has been documented in large cross-sectional studies [66,67,68]. This is likely because early adolescence and puberty are characterized by a temporary decrease in insulin sensitivity and an increase in insulin resistance [69], which can affect lipid metabolism and lead to increased blood cholesterol and TG, as well as decreased HDL-C levels [70].

The median BMI z-score increased from 0.19 to 0.30 between the pre- and post-pandemic periods. However, this increase did not reach statistical significance. In contrast, two meta-analyses [39,40] did find small but significant increases in BMI z-scores and in the prevalence of overweight and obesity among children and adolescents. However, heterogeneity was high for most outcomes in both studies, and the certainty of evidence was very low in the meta-analysis by Anderson et al. [39].

Interestingly, the increase in body weight and BMI was not significant in paediatric sub-populations with chronic conditions (T1DM and obesity) in the meta-analysis by Chang et al. [40], similarly to what was observed in our study sample. However, according to other studies, paediatric patients with overweight and obesity gained weight during the lockdowns [64,65,71,72]. Furthermore, the increase in BMI z-score and reduction in physical activity were more pronounced in children with obesity when compared to children with other endocrine diseases, despite no significant changes to dietary habits [73].

Epidemiological data have shown that increasing trajectories in adiposity in childhood [74] and adolescence [75] are associated with worse cardiometabolic profiles in adulthood. For a child with FH, who is already at a higher risk of CVD than their peers [76], overweight and obesity further increases that risk [77,78]. On the other hand, evidence suggests that reversing high BMI may reduce CVD risk [75,79]. This underscores the importance of nutritional management in paediatric FH, especially when overweight or obesity are present. In the present study, four participants who were previously at a normal weight became overweight during the pandemic. Whether or not this was a direct consequence of the COVID-19 lockdowns, it is crucial to provide closer monitoring and personalized nutritional intervention to these participants.

Another meta-analysis showed improvements in glycaemic control of paediatric patients with T1DM during lockdowns [80]. The authors hypothesized that home confinements may have allowed parents to be more engaged in the management of their child’s condition. In turn, family engagement facilitates healthy dietary changes and is associated with better health outcomes of children with chronic illnesses [81,82,83]. We speculate that this may have been an important factor contributing to the lack of deterioration of post-pandemic lipid and anthropometric profiles in our study. This also suggests that patients from our Reference Centre were well educated regarding the importance of a healthy lifestyle in FH, even before the pandemic. Finally, COVID-19-related fear may have prompted these patients and caregivers to adhere to lifestyle recommendations to reduce the risk of becoming severely ill.

Having a genetic diagnosis of FH in our study sample was not associated with higher LDL-C levels at any time point or a worsening in lipid profile post-pandemic. There are many possible explanations for this. The fact that few participants had identifiable FH mutations likely limited our statistical power to detect significant associations. Additionally, most participants had not undergone genetic testing. Lastly, individuals with negative genetic results may have undetected pathogenic variants, variants in genes that were not analysed in the genetic tests or variants in as-yet-undiscovered genes [1]. Otherwise, the FH phenotype observed in these participants could be explained by a polygenic aetiology [19] or high levels of Lp(a) [84].

To the best of our knowledge, this was the first study to investigate the effects of the restrictive measures and lockdowns amid the COVID-19 pandemic on children and adolescents with FH. We believe this constitutes a major strength of this study. Additionally, this study included other biochemical parameters besides lipid profile and all variables were directly measured, which increased the reliability of our findings.

However, our study had several limitations. This study was retrospective and designed after the start of the COVID-19 pandemic. Data at baseline were not properly controlled, which resulted in lower quality data and more missing information compared to the post-pandemic period. It is possible that this may have masked the magnitude of changes in some relevant outcomes. A convenience sampling method was used, although recruitment of consecutive cases could have mitigated the risk of bias and lack of representativity. Furthermore, the study sample was small and significantly heterogenous in terms of age, length of follow-up, genetic profile and use of lipid-lowering drugs, thereby limiting our statistical power. Pubertal development and body composition were also not assessed. Finally, the absence of data on dietary and physical activity habits, adherence to prescribed drugs, and environmental factors in the pre-pandemic period restricts our ability to fully understand the influence of these lifestyle factors on the metabolic profile of paediatric patients with FH during lockdowns.

## 5. Conclusions

The results of the present exploratory analysis suggest that the lipid and anthropometric profiles of children and adolescents with FH did not deteriorate after successive lockdowns and restrictive measures imposed to contain the spread of COVID-19. These are positive results that contradicted our hypothesis that there would be some deterioration in lipid and anthropometric profiles. However, there was a small increase in the number of participants with overweight in the post-pandemic period, suggesting a possible negative effect of the lockdowns in these participants.

We speculate that the clinical team at the Reference Centre were successful in educating patients and families about FH, the associated cardiovascular risk and the need to maintain a healthy lifestyle and diet. Ultimately, this awareness may have contributed to patients maintaining healthy habits during the COVID-19 lockdown period.

However, due to the small and heterogeneous study sample, the results of this exploratory analysis should be interpreted with caution. Further research using a multicentre approach is essential to gain a better understanding of the impact of lockdowns on this population.

## Figures and Tables

**Table 1:** Baseline sample characteristics.

Baseline Characteristics	N (%) Median [P25, P75]
**Gender**	
Male	13 (43%)
Female	17 (57%)
**Age (years)**	11 [9, 13]
**Age at follow-up initiation (years)**	8 [5, 11]
**Time in follow-up (years)**	3 [1, 5]
**Pre-treatment LDL-C (mg/dL)**	158 [136, 197]
**Genetic testing (yes, n)**	12 (40%)
No mutations identified	6 (20%)
Pathogenic LDLR mutation	3 (10%)
LDLR variant with likely pathogenicity	3 (10%)
**Relatives with same FH mutation (yes, n)**	5 (17%)
**Family history of premature CHD/sudden death (yes, n)**	8 (27%)
**Family members with hypercholesterolemia (n)**	
0	3 (10%)
1–2	14 (47%)
≥3	13 (43%)
**On lipid-lowering medication (yes, n)**	8 (27%)
Statin only	6 (20%)
Rosuvastatin	4 (13%)
Pravastatin	2 (7%)
Statin + ezetimibe	2 (7%)

Data are presented as N (%) or median [P25, P75]. CHD, coronary heart disease; FH, familial hypercholesterolemia; LDL-C, low-density lipoprotein cholesterol; LDLR, LDL receptor gene.

**Table 2:** Comparison between baseline and post-pandemic regarding age, biochemical parameters and anthropometry.

	N	Baseline	Post-Pandemic	*p* *
Age (years)	30	11 [9, 13]	14 [12, 16]	-
**Biochemical parameters**				
LDL-C (mg/dL)	30	125 [112, 150]	125 [100, 147]	0.894
HDL-C (mg/dL)	30	58 [52, 65]	56 [51, 61]	0.107
TG (mg/dL)	30	64 [44, 86]	59 [42, 86]	0.178
TC (mg/dL)	10	197 [178, 228]	211 [157, 244]	0.919
Lp(a) (mg/dL)	11	21 [4, 47]	20 [6, 35]	0.341
apoA (mg/dL)	15	143 [136, 154]	141 [133, 161]	0.887
apoB (mg/dL)	14	88 [80, 96]	82 [68, 101]	0.490
Glucose (mg/dL)	16	92 [86, 94]	91 [88, 98]	0.178
ALT (U/L)	17	16 [14, 26]	16 [14, 20]	0.850
AST (U/L)	19	21 [20, 29]	21 [17, 24]	0.238
**Anthropometry**				
Weight (kg)	28	38.7 [28.2, 50.4]	50.9 [37.7, 58.7]	<0.001
Height (cm)	28	146.8 [130.5, 158.0]	158.4 [141.9, 166.0]	<0.001
Height z-score	28	−0.72 [−1.14, 0.56]	−0.54 [−1.17, 0.90]	0.097
BMI (kg/m^2^)	28	17.9 [16.8, 19.9]	20.3 [17.7, 21.7]	<0.001
BMI z-score	28	0.19 [−0.58, 0.89]	0.30 [−0.48, 1.10]	0.524

* Data are presented as medians [P25, P75]. * Wilcoxon signed-rank test was performed for continuous variables. ALT, alanine aminotransferase; apoA, apolipoprotein A; apoB, apolipoprotein B; AST, aspartate aminotransferase; BMI, body mass index; HDL-C, high-density lipoprotein cholesterol; LDL-C, low-density lipoprotein cholesterol; Lp(a), lipoprotein(a); TC, total cholesterol; TG, triglycerides.

## Data Availability

The data will be made available from the authors upon reasonable request. The data are not publicly available due to privacy and ethical reasons.
